# The sense of balance in humans: Structural features of otoconia and their response to linear acceleration

**DOI:** 10.1371/journal.pone.0175769

**Published:** 2017-04-13

**Authors:** Rüdiger Kniep, Dirk Zahn, Jana Wulfes, Leif Erik Walther

**Affiliations:** 1 Max Planck Institute for Chemical Physics of Solids, Dresden, Germany; 2 Computer Chemistry Center, Department of Chemistry and Pharmacy, Friedrich-Alexander University Erlangen-Nürnberg, Erlangen, Germany; 3 Department of Otorhinolaryngology, Head and Neck Surgery, University Medicine Mannheim, University of Heidelberg, Mannheim, Germany; University of South Florida, UNITED STATES

## Abstract

We explored the functional role of individual otoconia within the otolith system of mammalians responsible for the detection of linear accelerations and head tilts in relation to the gravity vector. Details of the inner structure and the shape of intact human and artificial otoconia were studied using environmental scanning electron microscopy (ESEM), including decalcification by ethylenediaminetetraacetic acid (EDTA) to discriminate local calcium carbonate density. Considerable differences between the rhombohedral faces of human and artificial otoconia already indicate that the inner architecture of otoconia is not consistent with the point group -3m. This is clearly confirmed by decalcified otoconia specimen which are characterized by a non-centrosymmetric volume distribution of the compact 3+3 branches. This structural evidence for asymmetric mass distribution was further supported by light microscopy in combination with a high speed camera showing the movement of single otoconia specimen (artificial specimen) under gravitational influence within a viscous medium (artificial endolymph). Moreover, the response of otoconia to linear acceleration forces was investigated by particle dynamics simulations. Both, time-resolved microscopy and computer simulations of otoconia acceleration show that the dislocation of otoconia include significant rotational movement stemming from density asymmetry. Based on these findings, we suggest an otolith membrane expansion/stiffening mechanism for enhanced response to linear acceleration transmitted to the vestibular hair cells.

## Introduction

**Fig 1:**
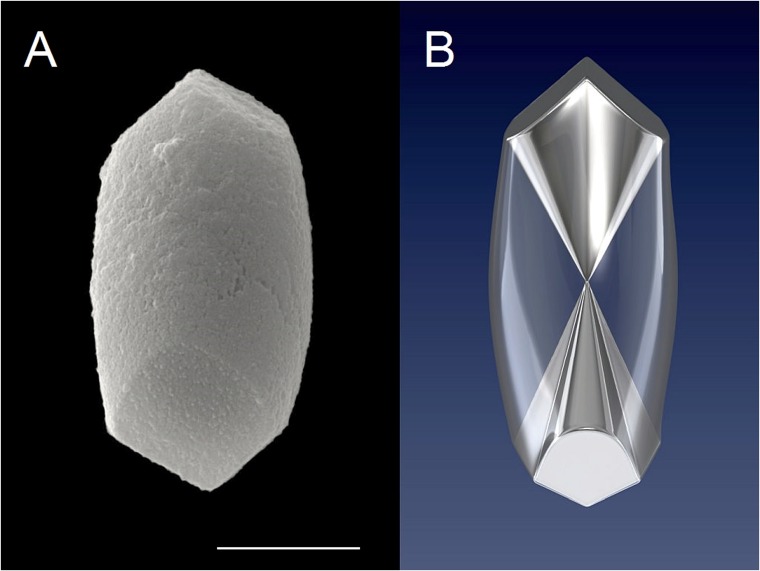
Outer shape and inner structure of human otoconia. *(A)* Intact human otoconium showing the bulbous (cylindrical) belly and the terminal rhombohedral faces representing the visible part of the 3+3 branches inside the volume. ESEM (Environmental scanning electron microscope)-image, high vacuum mode (HV), 15 kV. *(B)* 3D visualization of the inner architecture of an otoconium, keeping point group -3m for calcite. Less dense belly area (transparent) and 3+3 dense (compact) branches starting from the center of symmetry. Scale bar *(A)*: 5 μm.

The mammalian inner ear realizes its function by means of the mechanical activation of vestibular and auditory hair cells. Vestibular stimuli are measured by five paired vestibular sensors: Three semicircular canals for detection of angular acceleration, and the otolith system consisting of the utricle and the saccule, representing linear motion sensors. Utricle and saccule each contain thousands of otoconia embedded in an acellular organic matrix (otoconial complex) [1,2]. This complex provides inertial forces to stimulate the underlying vestibular hair cells for the detection of translational head stimuli caused by linear accelerations and head tilts in relation to the gravity vector [3,4]. Mammalian otoconia with characteristic shape (Fig 1) are arranged to form various layers [5]. Their mean size is about 10 μm, although there is a specific size distribution of otoconia over the otolithic membrane, which, however, is not in the focus of the present investigation [6]. Mammalian otoconia are calcite-based nanocomposites containing a small amount (<5 wt. %) of protein molecules [7–9]. These proteins, such as otoconin 90 and otolin are not only integrated into the composite structure of otoconia but also grow out of otoconial volume to form fibrils, which interconnect otoconia within a flexible network [3,9–11].

Biomimetic (“artificial”) otoconia (calcite gelatin nanocomposits, CGC) can be obtained by double-diffusion growth in gelatin-gel matrices [7]. Except for their bigger size (50–400 μm), artificial otoconia reveal the same chemical and structural properties as human (mammalian) otoconia [7–9]. Biomimetic otoconia have already been used as a suitable model system for the investigation of structural alterations caused by chemical attacks and ototoxic medications (gentamicin) [2,12].

Several models (e.g. finite element and difference models) have been applied in order to simulate movement-reactions of the otolithic membrane caused by translational and gravitational effects.

Dai et al. presented a model of otolith function based on experimental data hypothesizing otoconial displacement using the density of “a layer of otoconia” (calcite) and the “gelatinous mass” (matrix proteins) [13]. Grant et al. reported on algorithms to calculate the contributions of elastic, viscous and inertial terms [14]. Up to now, there has been, to our knowledge, no investigation into the function of otoconia in relation to their specific inner architecture, which is the result of structure development during evolution. In the case of mammals, the calcite modification of calcium carbonate is established as the inorganic component of the otoconial composite which, in contrast to aragonite and vaterite (present in otoliths of fish and amphibians), has the structural advantage of not preferring a specific growth direction which can lead to the formation of rods and/or needles [15]. Thus the organic components (proteins), which take over control of the shape development of otoconia, can more easily interact with the inorganic nano-units of calcite, to form complex architectures, which perfectly match the required response (function).

At first glance, the outer shape of (human) otoconia appears to be consistent with an overall symmetry close to the point group -3m. Otoconia are built up of 3+3 dense branches starting from the otoconial center (3-fold inversion axis) and ending in rhombohedral faces. The density distribution within a single otoconium is given by a cylindrical, less dense (porous) belly area and the 3+3 dense (compact) branches resulting in inhomogeneous (centrosymmetric) arrangement, just like a pendulum (see Fig 1) [5,7–9]. This kind of strict structure description based on the point group -3m was supported not least by the fact that single otoconia give X-ray (Bragg-) patterns like calcite single crystals [5,7–9]. On the other hand, the anisotropic growth of otoconia (branches grow quickly and the belly region grows with a significant delay), together with the resulting composite nanostructure can be taken as a hint to call the strict -3m description into question. In fact, detailed investigations of the outer shape of otoconia lead to the conclusion, that the rhombohedral faces are different in size, thereby destroying the centrosymmetry, which, at the same time, affects the density distribution within the volume [16]. This observation is taken as a first suggestion, that deviations from centrosymmetry are prerequisites for the function of otoconia belonging to the otoconial complex.

In this study we carefully investigated outer shape and inner structure of human and artificial otoconia and performed dynamic experiments using artificial (biomimetic) otoconia, in viscous artificial endolymph under the influence of gravity. In addition, we performed particle dynamics simulations in order to contrast symmetrical and asymmetrical mass distribution in otoconia during linear acceleration.

## Materials and methods

### Ethics

The present study was conducted in conformity with the declaration of Helsinki 1975, revised in 1983, and approved by the Ethics Committee of the University Medicine Mannheim, University of Heidelberg (2012-612N-MA). Written informed consent was obtained from all participants after the experimental procedure was explained.

### Inner architecture of human and artificial otoconia

Vital human otoconia were extracted as described recently [9]. Specimens of otoconia were characterized by environmental scanning electron microscopy (ESEM, FEI Quanta 200 FEGi, Eindhoven, Netherlands) in low vacuum (LV, 60 Pa) and high vacuum (HV, 2x10^−4^ Pa) modes. Optionally, a backscatter and a secondary electron detector were used in order to obtain increased contrast differences. The morphology (outer shape) of a number (n = 1000) of human and artificial otoconia (calcite gelatin nanocomposites, CGC, n = 100) was carefully investigated by means of ESEM.

For clarification of the inner architecture, decalcification of human otoconia was performed by treatment with ethylenediaminetetraacetic acid (EDTA, c = 0.001–0.0025 mol/l). After step-wise dissolution of the less dense, porous belly areas, the 3+3, more compact branches became visible. In this way direct correlation between the size of a rhombohedral face and the volume of the respective branch was obtained.

Artificial otoconia (CGC) were grown by double diffusion into a gelatin gel (denatured collagen) according to the methods described before [7,8]. The gelatin gel was taken not only as the diffusion matrix but was also incorporated into the system forming the nanocomposite.

### Positional changes of biomimetic otoconia within a tube of viscous artificial endolymph

For observation of positional changes of otoconia under gravity influence, single artificial specimen (CGC) were used. Artificial otoconia are bigger in size than human species. This allows for more precise microscopic observation of biomimetic particles. In this connection it is important to state that for dynamical processes upon acceleration (gravity influence) sizes are scalable and thus do not affect the mechanistic process. Single specimens of randomly chosen (n = 50) artificial otoconia were placed in a tube of viscous artificial endolymph. The statistical variance of the volume asymmetry in different otoconia was not investigated in detail. Our experience with several hundreds of biogenic and biomimetic specimens, however, allows the statement that by taking into account the dissolution experiments and the estimated differences in branch volumes (ratio of "top three" to "bottom three" branches along an idealized 3-fold axis) it goes down to about 1:0.4.

Since artificial endolymph, as reported by Marcus et al. [17], leads to chemical interactions with otoconia and does not contain any organic component as is present in the otoconial membrane, we used an artificial endolymph as follows: Aqueous solution of 90 mmol/l potassium chloride together with 0.8 wt.% gelatin. The liquid (15 ml) was then filled into vertically placed glass tubes (diameter 20 mm, length 23 mm). A Nikon zoom stereomicroscope (SMZ 1500, Nikon Instruments Europe B.V., Germany) in combination with a ProgRes SpeedXT core 5 camera (Jenoptik, Germany) was used for observation and recording of live image series during particle movement under gravitational conditions.

### Particle dynamics simulations by use of otoconia models with symmetric and asymmetric mass distributions

Particle dynamics simulations were performed on the basis of the molecular dynamics simulation package [18]. A generic coarse-grained model was employed to study a 100×50×30 μm sized cell encompassing a fluid and an otoconium. The latter was bound to a substrate surface, comprising impermeable walls confining the x- and y-direction, whilst periodic boundary conditions were used in the z-direction.

The models are considered as generic and provide only qualitative insights, the only quantitative aspect reflects the direct comparison of symmetric and asymmetric mass distribution in the otoconia. The fluid model, 12,500 soft sphere particles, were described by a Lennard-Jones potential. The otoconium was represented by a rigid unit of three linearly arranged spheres, which was bound to a substrate via two polymer strains each comprising 9 beads connected by harmonic springs. For all particles, the same van-der-Waals binding energy was chosen, whilst the van-der-Waals radii of the otoconium building blocks, the polymer beads and the fluid model reads 5, 4 and 2 μm, respectively. The overall mass of the otoconium was chosen to reproduce a density ratio of 2 with respect to the fluid model. In independent simulation setups, symmetric mass distribution was contrasted to asymmetric mass distribution (25% higher density assumed for the one end, accordingly 12.5% decrease for the two other building blocks). Acceleration was applied parallel and perpendicular to the long axis of the otoconium, leading to a total of 4 different simulation setups.

## Results

**Fig 2:**
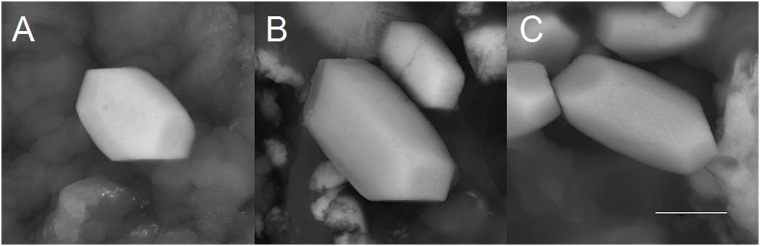
Variations in the size of the terminal faces of human otoconia. *(A*,*B*,*C)* Vital human otoconia show an asymmetric size distribution of the terminal faces indicating that there is non-centrosymmetric mass distribution parallel to the long axis of each single otoconium. ESEM, LV, low vacuum, 15 kV. Scale bar in (*A*,*B*,*C)* 3 μm. Scale bar in *(C)*, also for *(A*,*B)*: 3 μm.

**Fig 3:**
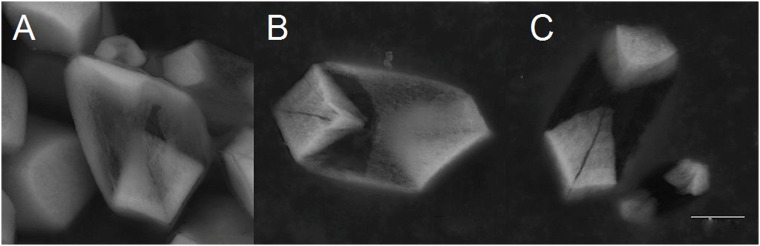
Human otoconia after treatment with EDTA (c = 0,107mol/l). *(A*,*B*,*C)* Dissolution of the belly area. The more dense non-centrosymmetric branches remain as residues. The technique of partial dissolution of the calcite component gives insight into the inner structure of otoconia and clearly reveals an asymmetric mass distribution within single otoconia specimens. ESEM, LV, low vacuum, 15 kV. Scale bar in *(C)*, also for *(A*,*B)*: 3 μm.

**Fig 4:**
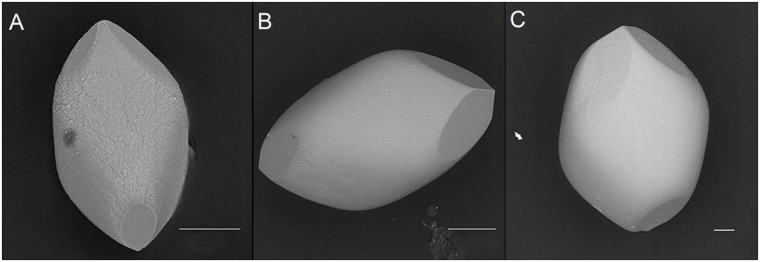
Biomimetic otoconia (Calcite Gelatin Composits, CGC) with different sizes of their rhombohedral faces. *(A*,*B*,*C)* As a consequence the mass-distribution parallel to the long axis of specimen is non-centrosymmetric (see Fig 4). ESEM, LV, low vacuum, 15 kV. Scale bar in *(A)* and *(B)*: 50μm. Scale bar *(C)*: 100 μm.

**Fig 5:**
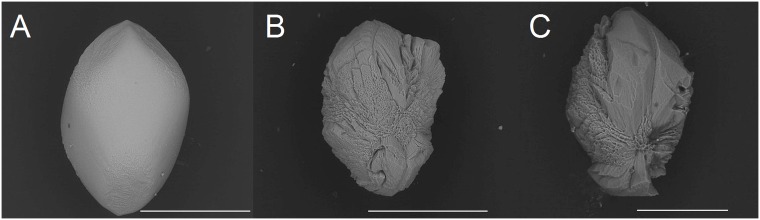
Biomimetic otoconia (Calcite Gelatin Composit, CGC) before and after treatment with EDTA (c = 0,107mol/l). *(A*,*B*,*C)* The preferred dissolution of the belly area clearly reveals non-centrosymmetric mass distribution of the branches (top and bottom along an idealized 3-fold axis). ESEM, LV, low vacuum, 15 kV. Scale bar in *(A)*: 500 μm, in *(B)*: 200μm and in (C): 100 μm.

**Fig 6:**
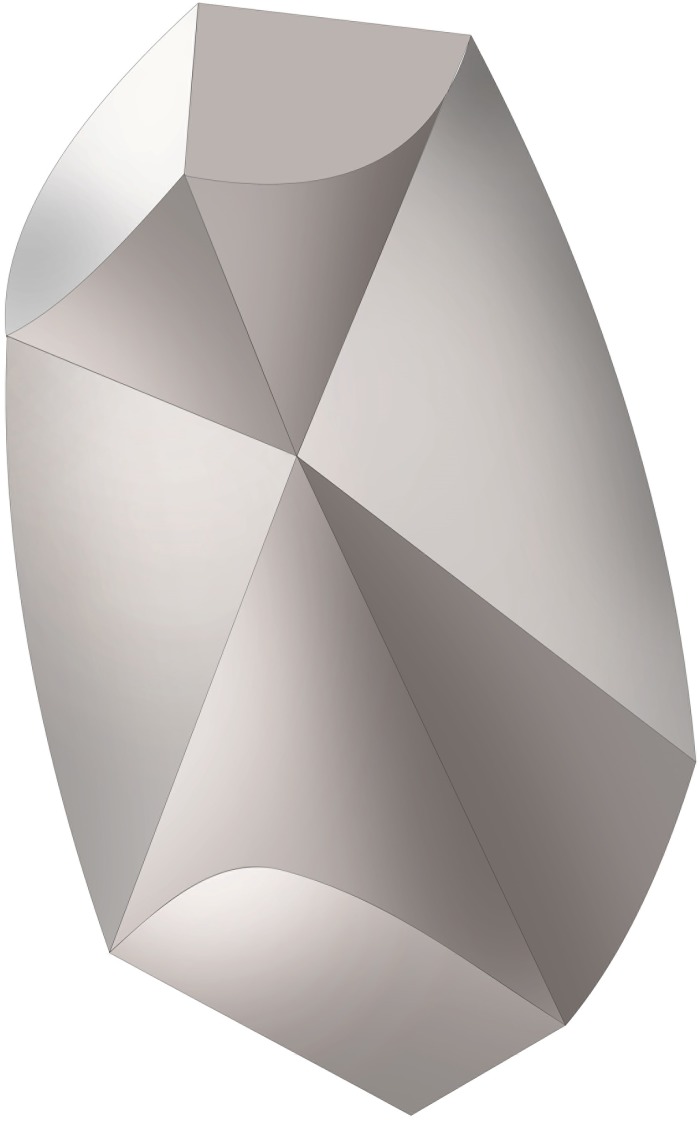
3 D-model of the inner structure of a single human otoconium. Differences in size among the rhombohedral faces clearly correlate with variations in volume among the 3+3 branches. The inner structure deviates from centrosymmetry indicating non-centrosymmetric mass distribution parallel to the long axis of the otoconium.

ESEM investigations of human and biomimetic otoconia revealed that non-centrosymmetric mass distribution is the predominant feature (Figs 2–5). Variations in size among the rhombohedral faces clearly correspond to variations in volume among the branches causing asymmetric mass distribution. By stepwise (partial) decalcification with EDTA, the inner architecture of human otoconia becomes clearly visible (Fig 3). Differences in size among the rhombohedral faces correlate with variations in volume among the 3+3 branches. Thus, the inner structure deviates from centrosymmetry indicating non-centrosymmetric mass distribution parallel to the long axis of the otoconium shown in a model of a single otoconium (Fig 6).

**Fig 7:**
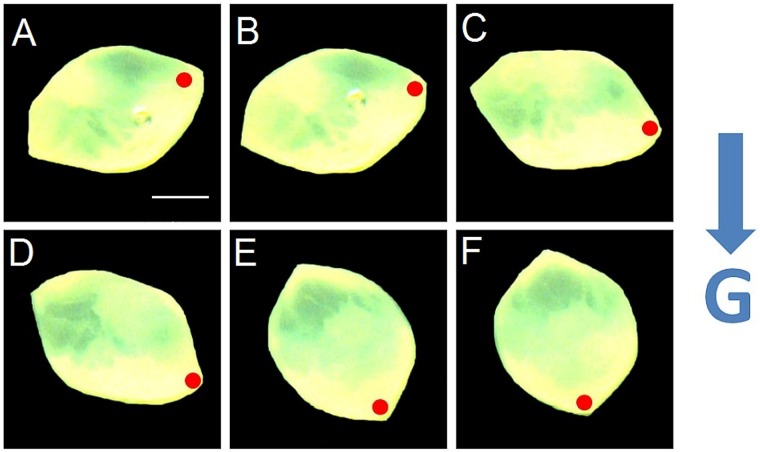
Biomimetic otoconium within a tube of artificial endolymph under the influence of gravity. *(A-C)* Directional changes in time steps of 3 sec indicating the movement of the heavier mass fraction (red point) in the direction of the gravitational vector (G), indicating the non-centrosymmetric mass distribution. CGC, size about 700 μm, recorded by means of a Nikon SMZ 1500 stereomicroscope in combination with a ProgRes SpeedXT core 5 camera during sinking (about 20 sec.) in the liquid medium (artificial endolymph). Scale bar in *(A)* also for *(B-F)* 200 μm.

Microscopic investigation of artificial otoconia under gravitational conditions within a tube of viscous liquid (artificial endolymph) revealed a turn of the specimen during sinking with the heavier end towards the gravitational vector. In simple terms, the behavior of the otoconia resembles that of a buoy (Fig 7).

**Fig 8:**
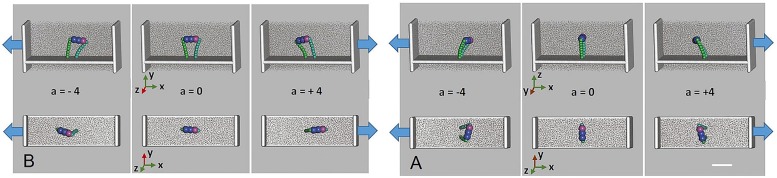
Simulation model of a single asymmetric otoconium represented by a rigid unit of 3 linearly arranged spheres (blue, purple). *(A*,*B)* Two independent simulation setups were dedicated to the purpose of studying acceleration parallel *(A)* and normal *(B)* to the long axis of the otoconium fixed to the substrate layer. The otoconium is bound to a substrate layer via two polymer chains (green), each mimicked by a series of harmonic springs. The overall system is subjected to periodic boundary conditions along the y and z directions, respectively. Acceleration (blue arrows) is applied along the x-direction, resulting in a density gradient of the model liquid (grey dots). Because of its larger specific weight, the otoconium moves into the opposite direction. Note the tilting of the otoconium resulting from its asymmetric mass distribution (assumed as 20% larger for the bottom end, and 12.5% less for the other two building blocks as colored in purple and blue, respectively). Scale bar in *(A)* also for *(B)* 10 μm.

Computer simulations show two distinct effects of imposing acceleration on the confining walls of the fluid model comprising otoconia (Fig 8A and 8B). The inertia of the mobile species. i.e. the fluid and (partially) the otoconium leads to flow/movement to establish a density gradient. When maintaining constant acceleration, flow decreases and a constant density gradient is reached. As a consequence of the different fluid and otoconia densities, a roughly constant shifting/tilting of the otoconia is observed. In the case of symmetric otoconia specimens uniform displacement is observed, whilst a significant tilting is found when accelerating the non-centrosymmetric otoconia.

## Discussion

Our experiments clearly show an asymmetric mass distribution of the otoconia (see Figs 2–5). In general, such breaking of symmetry is remarkable, and indeed the required biassing can only prevail in nature if it is related to an evolutionary benefit. In the case of human otoconia, the close interaction of fiber-proteins with calcite nanoparticles causes their peculiar inner architecture to be dominated by mesoscopic structuring, which "formally" is not consistent with the fact that single otoconia show a Bragg-diffraction pattern in the same way as a single crystal. Nature just creates a higher level of complexity. We thus expect the asymmetric mass distribution of the otoconia to play a functional role for the otoconial membrane.

To explore the underlying mechanisms, we performed particle dynamics simulations based on simplified model systems by use of single otoconial specimens connected to a substrate layer via two polymer strands grown out of the two ends of the otoconium (see Fig 8A and 8B). The preferred and orientated growth of the protein fibrils out of the rhombohedral faces of an otoconium and the interconnection of otoconia by polymer strands have only recently been shown for human specimen [8,9]. With this, the otoconial membrane can be considered as a network of layers (probably not more than three layers) of interconnected otoconia within a confined space and affecting each other in the course of positional changes. Detailed knowledge of otoconia fixation to the substrate containing the hair cells, acting as motion sensors, is not established, but is assumed to be realized by the fibrils growing out of their rhombohedral faces which then interconnect neighboring otoconia and also contribute to the fibril-network within the volume of the endolymph. However, it appears reasonable to assume, that only the lower layer of otoconia is directly attached to the substrate. In order to fix the single otoconium during our particle dynamics simulations it was attached to the substrate as described above.

**Fig 9:**
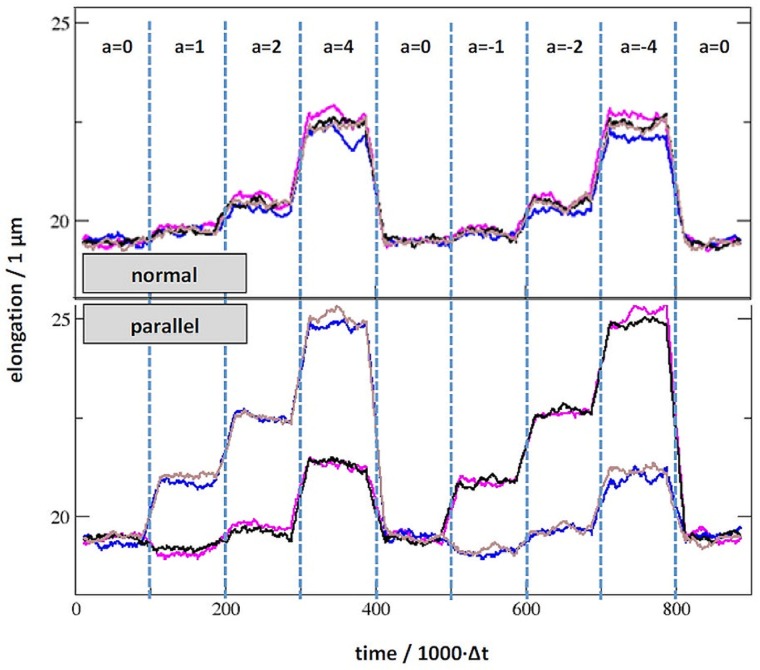
Elongation of the polymer strands resulting from acceleration normal and parallel to the orientation of otoconium fixation to the substrate. The simulation (see also Fig 7) encompasses the dynamics at equilibrium (a = 0), acceleration to a = ±1, a = ±2, a = ±4 (× 9.81 m∙s-2) and relaxation to a = 0 within subsequent runs of 100.000 time frames each and illustrates responses of the otoconium when arranged parallel and normal to the direction of acceleration. The purple line indicates elongation of the polymer strand which is attached to the denser end, whilst the blue curves refers to the polymer fixating the opposite end of the otoconium. For comparison, responses to a perfectly symmetric otoconium are illustrated by the black and brown curves, respectively.

To discriminate clearly the effect of asymmetric mass distribution, we performed analogous simulation runs for symmetric and asymmetric situations (Figs 8A, 8B and 9), and were able to distinguish clearly two modes of action during acceleration. The most obvious one is the overall displacement of the otoconium. This shift was identified for the two setups. However, significant tilting by up to 20° out of the starting position parallel to the substrate was observed for the asymmetric otoconium. In other words, the specific effect of the asymmetric mass distribution is rotation rather than the center of mass-displacement.

What could be the benefit of this rotation? The otoconial membrane as a collective of thousands of randomly orientated and interconnected otoconia (acting as receptor system) is in close contact to the vestibular hair cells (acting as mechanosensors) which send signals to the neurons upon deflection.

**Fig 10:**
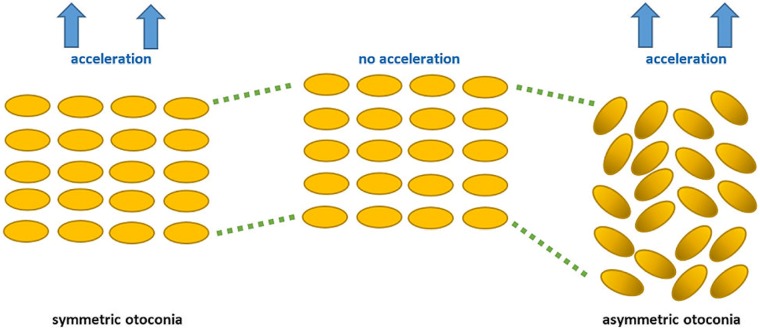
Displacement and rearrangement of symmetric and asymmetric otoconia in a simplified otolith membrane upon linear acceleration. Left: Membranes with symmetric otoconia show homogeneous displacement patterns according to the density gradient along the direction of acceleration. Right: Otoconia of asymmetric mass distribution (heavier ends shown in darker color) experience tilting/rotation in addition to dislocation. Note that the random pattern of clockwise/counter-clockwise tilting causes defects in the initially ordered matrix. While the pristine matrix reflects a more dense packing, defect formation demands additional volume and thus leads to an expansion/stiffening of the otolith membrane along the direction of acceleration. This affects the coupling of the otoconia-matrix with vestibular hair cells and leads to an increased signal strength being transmitted. The blue arrows show the direction of acceleration, whilst the green lines indicate the displacement of otoconia with respect to the otolith membrane in absence of acceleration (center).

Let us consider a simplified otoconial membrane (only one layer containing otoconia in parallel orientation to the substrate and with random distribution of their heavier ends) as shown in Fig 10. Depending on the nature of the interconnecting protein fibrils (polymer strands), the otoconial membrane is characterized by elasticity. During acceleration, the center of mass-displacement can be observed for symmetric and asymmetric otoconia (Fig 10). In addition, otoconia tilting (rotation) takes place in the asymmetric case, causing expansion of the elastic membrane and stiffening. The stronger the acceleration, the more intense is the otoconia tilting (rotation). The process is fully reversible and in the absence of acceleration forces the otoconia tilting (rotation) diminishes and the membrane gets back its former elasticity. Rotation of otoconia causes immediate effects on movement activities of neighboring (interconnected) otoconia which are all caught within confined space volumes. Thus expansion and stiffening of the otoconial membrane along the direction of acceleration leads to an increased signal strength which is directly transmitted by the underlying hair cells.

## Conclusions

Our experiments have shown that human and artificial otoconia reveal an asymmetric mass distribution. Deviation from centrosymmetry seems to be a prerequisite for detecting linear accelerations. From our experiments as well as the simulations it can be concluded that non-centrosymmetric otoconia architecture causes otoconia tilting (rotation), giving the flexible otolith membrane an extended movement spectrum for excitation of vestibular hair cells. Thus, a more distinctive membrane expansion along the direction of acceleration and stiffening allows a more differentiated stimulation of mechanoreceptors caused by 3D acceleration stimuli.

## Supporting information

S1 File Interim results of the simulation to reproduce the data used within this publication. (TXT)

S2 File Interim results of the simulation to reproduce the data used within this publication. (TXT)

S3 File Interim results of the simulation to reproduce the data used within this publication. (TXT)

S4 File Interim results of the simulation to reproduce the data used within this publication. (TXT)

S5 File Interim results of the simulation to reproduce the data used within this publication. (ZIP)

S6 File Interim results of the simulation to reproduce the data used within this publication. (ZIP)
